# Mutations in *MITF* and *PAX3* Cause “Splashed White” and Other White Spotting Phenotypes in Horses

**DOI:** 10.1371/journal.pgen.1002653

**Published:** 2012-04-12

**Authors:** Regula Hauswirth, Bianca Haase, Marlis Blatter, Samantha A. Brooks, Dominik Burger, Cord Drögemüller, Vincent Gerber, Diana Henke, Jozef Janda, Rony Jude, K. Gary Magdesian, Jacqueline M. Matthews, Pierre-André Poncet, Vilhjálmur Svansson, Teruaki Tozaki, Lorna Wilkinson-White, M. Cecilia T. Penedo, Stefan Rieder, Tosso Leeb

**Affiliations:** 1 Institute of Genetics, Vetsuisse Faculty, University of Bern, Bern, Switzerland; 2 DermFocus, University of Bern, Bern, Switzerland; 3 Faculty of Veterinary Science, University of Sydney, Sydney, Australia; 4 Swiss National Stud, ALP-Haras, Avenches, Switzerland; 5 Department of Animal Science, Cornell University, Ithaca, New York, United States of America; 6 Swiss Institute of Equine Medicine, Vetsuisse Faculty, ALP-Haras and University of Bern, Avenches, Switzerland; 7 Swiss Institute of Equine Medicine, Vetsuisse Faculty, University of Bern and ALP-Haras, Bern, Switzerland; 8 Division of Neurology, Vetsuisse Faculty, University of Bern, Bern, Switzerland; 9 Division of Experimental Clinical Research, Vetsuisse Faculty, University of Bern, Bern, Switzerland; 10 Certagen GmbH, Rheinbach, Germany; 11 Department of Medicine and Epidemiology, School of Veterinary Medicine, University of California Davis, Davis, California, United States of America; 12 School of Molecular Bioscience, University of Sydney, Sydney, Australia; 13 Institute for Experimental Pathology, University of Iceland, Reykjavík, Iceland; 14 Department of Molecular Genetics, Laboratory of Racing Chemistry, Utsunomiya, Japan; 15 Veterinary Genetics Laboratory, School of Veterinary Medicine, University of California Davis, Davis, California, United States of America; Stanford University School of Medicine, United States of America

## Abstract

During fetal development neural-crest-derived melanoblasts migrate across the entire body surface and differentiate into melanocytes, the pigment-producing cells. Alterations in this precisely regulated process can lead to white spotting patterns. White spotting patterns in horses are a complex trait with a large phenotypic variance ranging from minimal white markings up to completely white horses. The “splashed white” pattern is primarily characterized by an extremely large blaze, often accompanied by extended white markings at the distal limbs and blue eyes. Some, but not all, splashed white horses are deaf. We analyzed a Quarter Horse family segregating for the splashed white coat color. Genome-wide linkage analysis in 31 horses gave a positive LOD score of 1.6 in a region on chromosome 6 containing the *PAX3* gene. However, the linkage data were not in agreement with a monogenic inheritance of a single fully penetrant mutation. We sequenced the *PAX3* gene and identified a missense mutation in some, but not all, splashed white Quarter Horses. Genome-wide association analysis indicated a potential second signal near *MITF*. We therefore sequenced the *MITF* gene and found a 10 bp insertion in the melanocyte-specific promoter. The *MITF* promoter variant was present in some splashed white Quarter Horses from the studied family, but also in splashed white horses from other horse breeds. Finally, we identified two additional non-synonymous mutations in the *MITF* gene in unrelated horses with white spotting phenotypes. Thus, several independent mutations in *MITF* and *PAX3* together with known variants in the *EDNRB* and *KIT* genes explain a large proportion of horses with the more extreme white spotting phenotypes.

## Author Summary

White spotting coat color phenotypes are the result of aberrations in the development of melanocytes. The analysis of domestic animals with heritable white spotting phenotypes thus helps to better understand the complicated genetic network controlling the proliferation, migration, differentiation, and survival of pigment producing cells. We analyzed the so-called splashed white phenotype in horses, which is characterized by a very distinctive large blaze, extended white markings on the legs, and blue eyes. Splashed white horses are also frequently deaf. However, the phenotype is quite variable and, in some horses with minimal expression, the splashed white phenotype cannot be unambiguously discriminated from the “common” white markings. We studied horses from various breeds and found one mutation in the *PAX3* gene and two mutations in the *MITF* gene that cause the splashed white phenotype. A third mutation in the *MITF* gene, which we found in a single Franches-Montagnes horse, causes a new coat color phenotype, termed macchiato. Similar mutations in humans cause either Waardenburg or Tietz syndrome, which both are characterized by pigmentation defects and a predisposition for deafness. Our study reveals the molecular basis for a significant proportion of white spotting phenotypes that are intermediate between completely unpigmented horses and common white markings.

## Introduction

Coat color is a well-studied model trait for geneticists. Coat color phenotypes are relatively easy to record, which facilitates their analysis. In mammals melanocytes cover the entire body surface and are responsible for the pigmentation of skin, hairs, and eyes. Melanocytes are formed during fetal development from melanoblasts, which originate in the neural crest and migrate across the developing fetus in order to reach their final position on the body [1]. This developmental program requires a delicate level of regulation to ensure that the correct amount of cells reaches their final destination [2]–[5]. An over-proliferation of cells that have left their surrounding tissue might have fatal consequences for the developing fetus [6]. If however too few of the migrating melanoblasts survive, this will lead to partially or completely unpigmented phenotypes [7]. Domestic animals with such unpigmented phenotypes have been highly valued due to their striking appearance and have often been actively selected in animal breeding. Consequently, our modern domestic animals provide a large repertoire of spontaneous mutants that allow the dissection of the contributions of individual genes to migration, proliferation, differentiation, and survival of melanocytes.

From a genetic point of view, white spotting is considered a complex trait [8]. Phenotypes range from tiny white spots at the extremities of the body to large unpigmented areas, in symmetrical or asymmetrical patterns, up to completely unpigmented animals [9]. Different combinations of alleles at several loci can interact and it is generally impossible to precisely predict the genotype of an animal based only on a given white spotting phenotype.

Splashed white is a distinctive but nevertheless quite variable white spotting pattern in horses, which is primarily characterized by extensive depigmentation of the head. Splashed white horses often have blue eyes and they are sometimes deaf [9]. This phenotype has not yet been characterized at the molecular level.

Mutations in two genes have been identified to cause pronounced depigmentation phenotypes in horses thus far. A total of 19 different mutant alleles at or near the *KIT* gene were reported to cause either completely white horses or horses with pronounced depigmentation, such as the dominant white, tobiano, and sabino-1 spotting patterns [10]–[14]. The frame overo spotting pattern, which is phenotypically overlapping with the splashed white phenotype, is caused by a missense mutation in the *EDNRB* gene [15]–[17]. *EDNRB* mutations are also found in a small fraction of human patients with Waardenburg syndrome (WS). WS is characterized by pigmentation abnormalities, such as a typical white forelock or white skin patches, strikingly blue or heterochromatic irises, and varying degrees of sensorineural deafness and skeletal dysmorphologies. Other forms of human Waardenburg syndrome are caused by mutations in *EDN3*, *MITF*, *PAX3*, *SNAI2*, and *SOX10* [18]. Mouse mutants for these genes are available and show largely similar although not completely identical phenotypes [19].

In this study we report the identification of several independent mutations in horses with striking depigmentation phenotypes that show parallels to human Waardenburg syndrome.

## Results

### Splashed white in a Quarter Horse family

**Figure 1:**
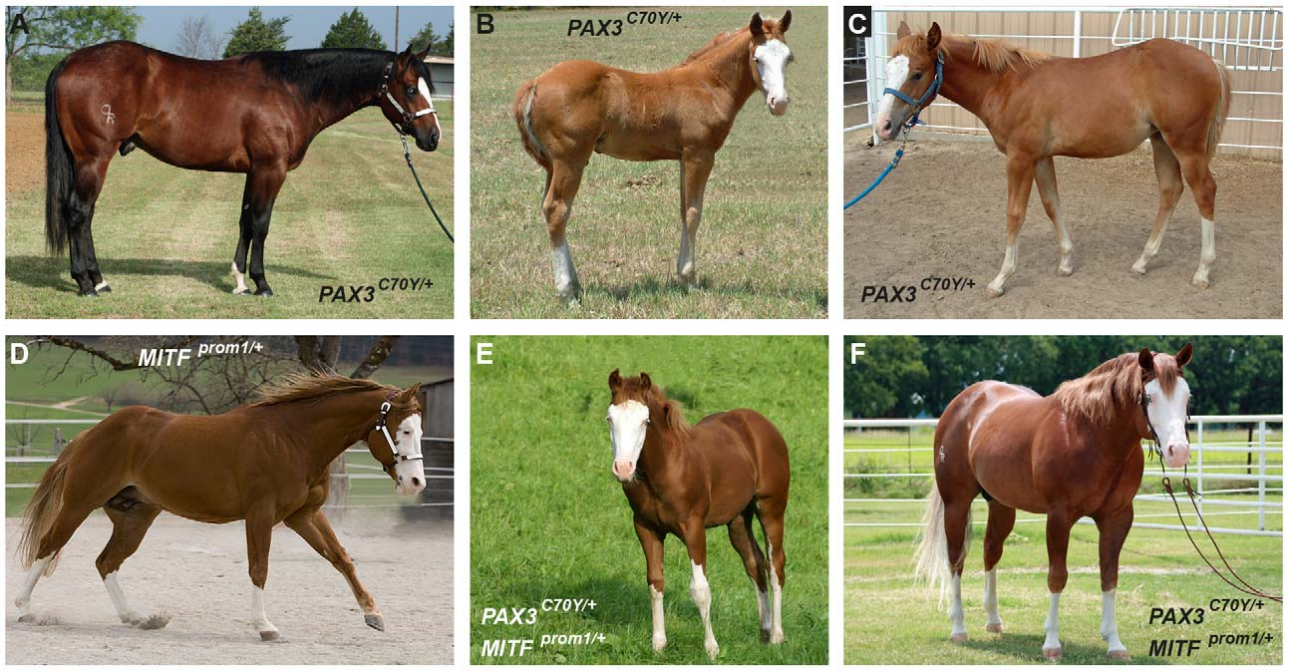
Phenotypes of splashed white horses from a Quarter Horse family. Note that the expression of the phenotype is quite variable. Splashed white may be caused by different mutations, even within closely related horses. (A) Splashed white bay horse. The unpigmented areas are relatively small, but the horse has blue eyes. (B) Typical expression of the splashed white phenotype in a chestnut horse with blue eyes. (C) A splashed white chestnut horse with normal eye color and a relatively small blaze. (D) Splashed white coat color and brown eyes in a chestnut horse. (E, F) Two horses carrying both the *PAX3^C70Y^* and *MITF^prom1^* alleles with typical splashed white phenotypes. Horses carrying one copy of either *PAX3^C70Y^* and/or *MITF^prom1^* show overlapping phenotypes. None of the horses in this figure carry the *EDNRB^I118K^* (overo) allele.

We obtained samples from a large Quarter Horse family segregating for a striking white spotting pattern termed splashed white (Figure 1). In our material this phenotype was characterized by a large blaze of variable size and shape. Many splashed white horses had an extremely large blaze extending over the eyes or even covering parts of the cheeks; such a head pattern is termed baldface. Splashed white horses also typically had high markings that extend high up on their legs and occasionally little white belly spots. Most of the splashed white horses had blue eyes or iris heterochromia. Although many splashed white horses have a very characteristic appearance, the phenotype is variable and in some splashed white horses the unpigmented areas are so small that we could not reliably distinguish these horses from horses with other subtle depigmentation phenotypes. Some, but not all of the sampled splashed white horses were deaf.

In this Quarter Horse family the splashed white coat color appeared to be inherited as an autosomal dominant trait. We therefore genotyped 24 cases and 7 controls from this family on the equine 50k SNP array and performed a linkage analysis (Figure S1). Parametric analysis with a fully dominant model of inheritance gave a maximum LOD score of 1.6 on ECA 6 with a corresponding maximum α of 0.78. These results suggested locus heterogeneity within the family. We also performed a case-control genome-wide association study (GWAS) with the SNP genotypes and found the strongest signal again on ECA 6 (p_raw_ = 5.6×10^−7^; p_genome_ = 0.003). The chromosomes with the next best associations were 11 and 16, each having SNPs associated at p_raw_ = 6.6×10^−5^, which is not genome-wide significant after permutation analysis (p_genome_ = 0.34).

The linked and associated region on ECA 6 contained *PAX3* as a strong functional candidate gene. We sequenced the nine exons of the *PAX3* gene in two cases and two controls and identified seven polymorphisms including one missense mutation, *PAX3*:c.209G>A (Table 1, Table S1). We then genotyped all remaining animals of the Quarter Horse family and other unrelated horses for this variant. We identified a total of 29 splashed white horses carrying the variant A-allele in heterozygous state. All these horses traced back to a female Quarter Horse born in 1987, whose genomic DNA from a hair-root sample tested homozygous wild-type. Thus, the mutation most likely arose in the germline of this animal. We did not detect the variant A-allele in 21 solid-colored Quarter Horses nor in 112 horses from 7 other breeds. We also did not find any horse with the homozygous variant A/A genotype (Table 2).

**Table 1:** Causative mutations for white spotting phenotypes.

Gene	Variant^a^	Variant (genomic)^b^	Allele designation	Allele designation (genetic testing)	Phenotype
*MITF*	n.a.	ECA16:g.20,117,302Tdelins11	*MITF^prom1^*	SW1	splashed white
*MITF*	c.837_841delGTGTC	ECA16:g.20,105,348_52del5	MITF^C280Sfs*20^	SW3	splashed white
*MITF*	c.929A>G	ECA16:g.20,103,081T>C	*MITF^N310S^*		macchiato
*PAX3*	c.209G>A	ECA6:g.11,429,753C>T	*PAX3^C70Y^*	SW2	splashed white

a numbering refers to NM_001163874.1 (*MITF*) and XM_001494972.3 (*PAX3*).

b build EquCab 2.0.

**Table 2:** Association of white spotting genotypes in different breeds.

	Gene	Genotype										
	*EDNRB*	*I118K/+*	*I118K/+*	*I118K/+*	*+/+*	*+/+*	*+/+*	*+/+*	*+/+*	*+/+*	*+/+*	No mutation found
	*MITF*	*+/+*	*prom1/+*	*prom1/prom1*	*prom1/+*	*prom1/prom1*	C280Sfs*20/+	prom1/C280Sfs*20	*+/+*	*prom1/+*	*prom1/prom1*	
	*PAX3*	*+/+*	*+/+*	*+/+*	*+/+*	*+/+*	*+/+*	*+/+*	*C70Y/+*	*C70Y/+*	*C70Y/+*	
Phenotype	Breed^a^											
Splashed white or other white spotting												
	QH/AP^b^	3	4	1	19	12	1	1	8	20	1	7
	HN	0	0	0	0	0	0	0	0	0	0	1
	IS	0	0	0	4^c^	7^c^	0	0	0	0	0	0
	MH	0	0	0	0	1	0	0	0	0	0	0
	OL	0	0	0	0	0	0	0	0	0	0	2
	SP	0	0	0	0	1	0	0	0	0	0	0
	TB	0	0	0	0	0	0	0	0	0	0	8
	TR	0	0	0	4	1	0	0	0	0	0	0
Solid colored												
	QH	0	0	0	0	0	0	0	0	0	0	21
	AS	n.d.	0	0	0	0	0	0	0	0	0	1
	FM	n.d.	0	0	0	0	0	0	0	0	0	96
	HF	n.d.	0	0	0	0	0	0	0	0	0	1
	IS	n.d.	0	0	0	0	0	0	0	0	0	4
	NO	n.d.	0	0	0	0	0	0	0	0	0	7
	TR	n.d.	0	0	0	0	0	0	0	0	0	1
	WB	n.d.	0	0	0	0	0	0	0	0	0	2

Results for the three newly described splashed white mutations and the overo mutation [15]–[17] are shown.

a QH, Quarter Horse; AP, American Paint Horse; HN, Hanoverian; IS, Icelandic Horse; MH, Miniature Horse; OL, Oldenburger; SP, Shetland Pony; TB, Thoroughbred; TR, Trakehner; AS, American Standardbred; FM, Franches-Montagnes; HF, Haflinger; NO, Noriker; WB, European Warmblood.

b Gene flow exists between QH and AP. Several horses in our study had double registrations with both the American Quarter Horse Association (AQHA) and the American Paint Horse Association (APHA).

c The Icelandic Horses with the splashed white or other white phenotype were not experimentally tested for the absence of the *EDNRB^I118K^*, MITF^C280fs*20^, and *PAX3^C70Y^* alleles. They were assumed to be homozygous wildtype at these positions.

**Figure 2:**
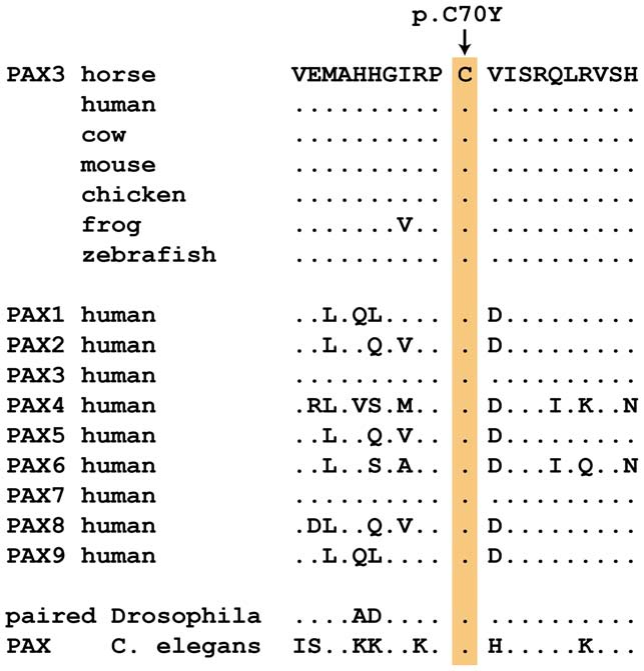
Region around the PAX3:p.C70Y mutation. The cysteine at position 70 is conserved in all known PAX paralogs.

The *PAX3*:c.209G>A variant is predicted to result in a non-conservative amino acid exchange p.C70Y in the so-called paired domain, which together with the homeobox domain mediates the DNA-binding of the transcription factor PAX3. The wild-type cysteine at this position is conserved across all known PAX paralogs in animals including *Drosophila* and *Caenorhabditis elegans* (Figure 2). Based on the X-ray structure of the paired domain of the human PAX6 protein, the side-chain of this cysteine is predicted to form a direct contact to the DNA backbone [20].

**Figure 3:**
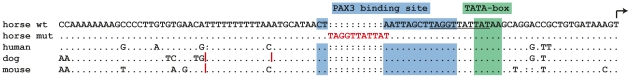
Proximal melanocyte-specific promoter of the *MITF* gene. The depicted region corresponds to positions −89 to +1 with respect to the transcription start site and is in reverse complementary orientation to the genomic reference sequence (ECA16:20,117,350–20,117,261; EquCab2.0). The three vertical red lines in the dog and mouse sequences represent small insertions. In some splashed white horses, an 11 bp motif shown in red replaces an adenine, which is part of a highly conserved PAX3 transcription factor binding site in the wild-type sequence. The inserted 11 bp sequence may have arisen by duplication from an identical sequence motif a few nucleotides further downstream (underlined).

The initial GWAS indicated potential secondary signals on ECA 11 and 16. We did not detect an obvious candidate gene on ECA 11. However, the *MITF* gene on ECA 16 represents a strong functional candidate for a white spotting phenotype. As the PAX3:p.C70Y variant did not explain the phenotype in all splashed white horses from the Quarter Horse family, we sequenced all exons and the known promoter elements from the *MITF* gene in two splashed white horses that did not have the *PAX3^C70Y^* allele and in two controls. We identified a total of 28 polymorphisms (Table S1). A variant in the proximal melanocyte-specific *MITF* promoter replacing a thymine with 11 nucleotides stood out as clear candidate for a non-coding regulatory mutation (ECA16:g.20,117,302Tdelins11; Figure 3). This variant interrupts a highly conserved binding site for PAX3 [21]–[23] and is located close to the mutation causing white spotting in dogs [24]. The variant allele was absent from 21 solid-colored Quarter Horses and from 112 horses with minimal white markings from 7 additional breeds. We found 19 splashed white horses that carried only the *MITF^prom1^* allele and 20 splashed white horses that carried both the *MITF^prom1^* and *PAX3^C70Y^* allele (Table 2).

**Figure 4:**
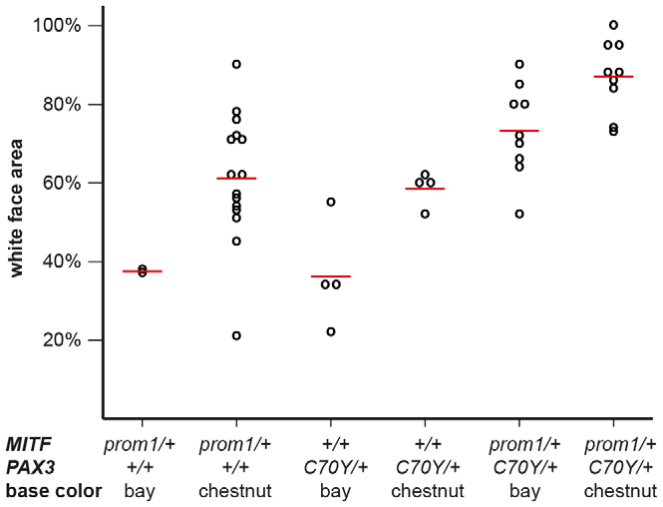
White face area in splashed white Quarter Horses with specific genotypes. Raw phenotypes for individual horses are indicated as black circles. The average values for each genotype class are indicated by red lines. While there is considerable variation in the phenotypic expression, the average proportion of white face area is higher in chestnut horses versus bay horses and it is also higher in horses carrying both the *MITF^prom1^* and the *PAX3^C70Y^* allele than in horses carrying only one of the two splashed white alleles (p<0.05, two-sided Welch t-test). The white face areas of all horses and the p-values between all different genotype classes are given in Table S2.

We quantitatively analyzed the proportion of depigmented skin in the face area of splashed white Quarter Horses in relation to their underlying *MITF* and *PAX3* genotypes as well as their base coat color (Figure S2, Figure S3). This analysis indicated that one copy of either the *MITF^prom1^* or the *PAX3^C70Y^* allele has a similar effect on the face pigmentation. The presence of both the *MITF^prom1^* and the *PAX3^C70Y^* alleles leads to a slightly more pronounced depigmentation of the face on average than either splashed white allele alone. This analysis also showed that the average white face area is more extended in splashed white chestnut horses compared to bay horses with the same splashed white genotype (Figure 4; Table S2).

### Breed distribution of the *MITF^prom1^* allele

We sequenced the coding regions and proximal promoter elements of the functional candidate genes *KIT*, *MITF*, and *PAX3* in other horses with white spotting phenotypes in additional horses from various breeds. We then noticed that the *MITF* promoter polymorphism described above is much more widespread across breeds than we had originally hypothesized. We found the insertion allele in 58 Quarter Horses and American Paint Horses with either the splashed white or more pronounced other white spotting phenotypes (see below). We also found this allele in five Trakehner horses with white spotting phenotypes. A detailed pedigree analysis revealed that all these horses are descendants from the Thoroughbred stallion Blair Athol born in 1861. This stallion also had a very large white blaze (Figure S4). The *MITF^prom1^* allele was also present in a Miniature Horse, a Shetland Pony, and 11 Icelandic Horses with either splashed white or more pronounced other white spotting phenotypes. Thus, the *MITF^prom1^* allele is probably several hundred years old and arose before the foundation of the modern horse breeds.

### More pronounced other white spotting phenotypes: Combinations of splashed white alleles

In our study we defined horses with ≥20% white face area and ≤10% white area on the body as “splashed white horses”. If a horse had >10% white body area, it was considered to have a more pronounced “other white spotting” phenotype. We noticed that several horses with more pronounced “other white spotting phenotypes” were offspring from two “splashed white” parents.

**Figure 5:**
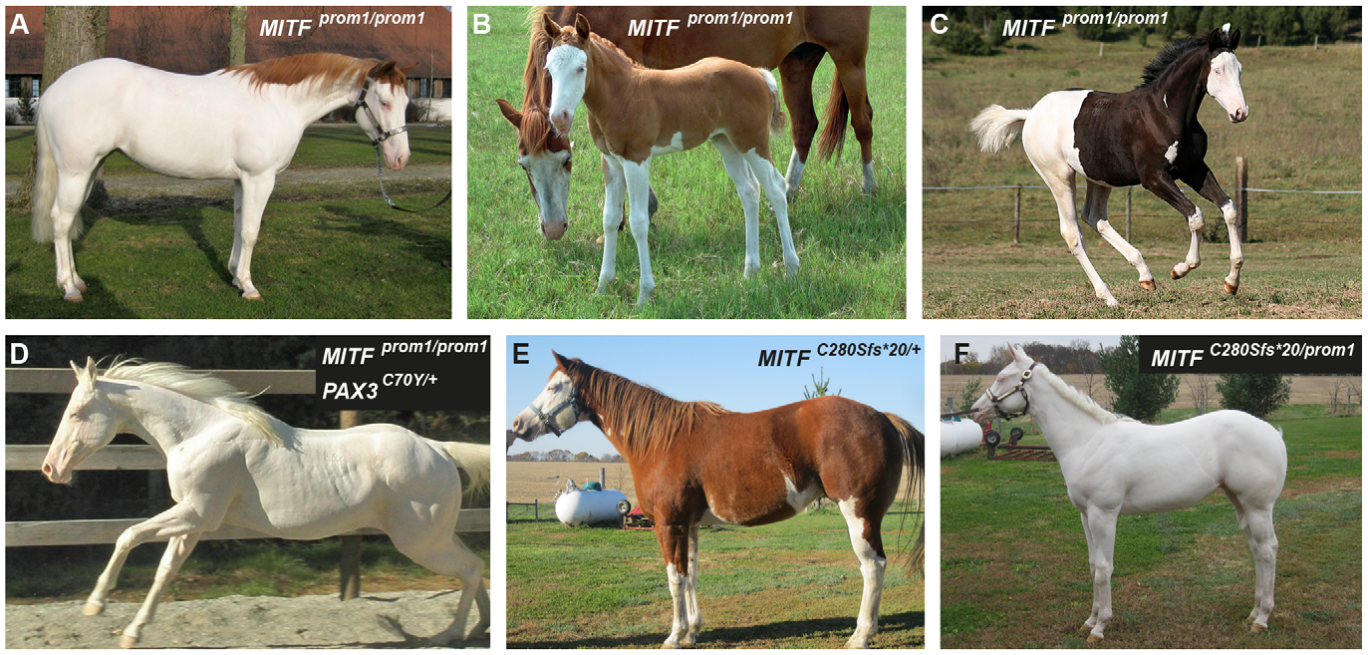
Phenotypes of horses with different combinations of splashed white alleles. (A) American Paint Horse with a very pronounced depigmentation phenotype. In addition to being homozygous for the *MITF^prom1^* allele, it also carries a private allele at the *KIT* gene (p.H40Q), which may enhance the depigmentation phenotype. The functional significance of the p.H40Q variant is unclear at the moment. (B) This Quarter Horse is also homozygous for the *MITF^prom1^* allele, but has substantially more residual pigmentation than the horse shown in panel A. (C) A Trakehner horse homozygous for the *MITF^prom1^* allele. (D) A completely white horse with multiple splashed white alleles. (E) Quarter Horse with the rare MITF^C280Sfs*20^ allele. This horse has a very pronounced splashed white phenotype with blue eyes and a largely unpigmented head and belly. (F) A compound heterozygote for two different *MITF* mutant alleles is completely white. None of the horses in this figure carry the *EDNRB^I118K^* (overo) allele.

We identified a total of 24 horses from 5 breeds that were homozygous for the *MITF* promoter variant. All these *MITF^prom1/prom1^*horses showed very pronounced but still variable depigmentation phenotypes. They had at least a white belly in addition to white legs and the white head (Figure 5A–5C). One of these horses had only small pigmented areas along the dorsal midline (Figure 5A). One horse homozygous for the *MITF^prom1^* allele and heterozygous for the *PAX3^C70Y^* allele was completely white and also deaf (Figure 5D).

In our mutation analysis we identified one horse with a splashed white phenotype including a white belly, which was wild-type for both the *MITF^prom1^* and the *PAX3^C70Y^* alleles (Figure 5E). This horse carried a small deletion in exon 5 of the *MITF* gene (c.837_841del5). The variant leads to a frameshift and a severely truncated *MITF* protein (p.C280Sfs*20), which might act in a dominant negative fashion. A completely white offspring of this horse was a compound heterozygote for the *MITF^prom1^* and the MITF^C280Sfs*20^ alleles (Figure 4F). The MITF^C280Sfs*20^ allele was absent from 21 solid Quarter Horses and 112 control horses from 7 additional breeds (Table 2).

In a few unrelated horses with large blazes we did not find any candidate causative mutations in the *KIT*, *MITF*, or *PAX3* candidate genes. These horses comprised 7 Quarter Horses, 1 Hanoverian, 2 Oldenburger, and 8 Thoroughbreds (Table 2).

### Macchiato in a Franches-Montagnes Horse

**Figure 6:**
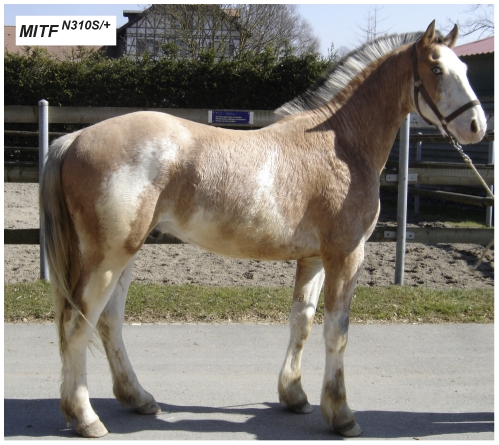
Macchiato coat color phenotype in a Franches-Montagnes stallion. Note the pronounced depigmentation on the body in addition to the large white blaze on the head, the white legs, and the blue eyes. This horse has a bay basic coat color.

In 2008 a colt with a striking white-spotting phenotype was born out of two solid-colored bay Franches-Montagnes parents. The coat color resembled a combination of white-spotting and coat color dilution (Figure 6). The parentage of this colt was experimentally verified using 13 microsatellite markers. Therefore, we assumed the coat color of this colt to be the result of a spontaneous *de novo* mutation and subsequently termed it “macchiato”. In 2010, we performed a detailed clinical and spermatological examination, which revealed that the two year old macchiato stallion was deaf and had a low progressive sperm motility.

**Figure 7:**
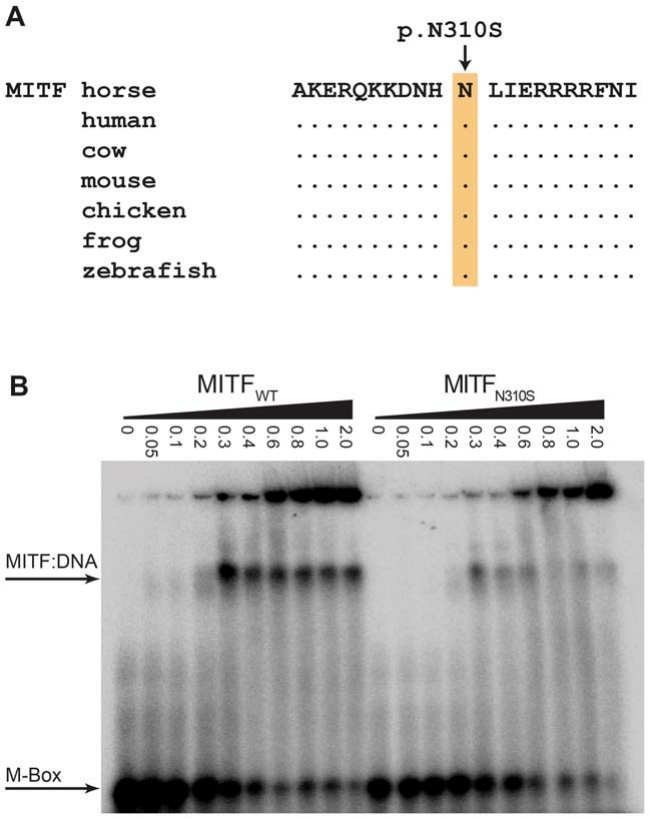
Functional validation of the MITF:p.N310S mutation. (A) The basic DNA binding domain in the vicinity of the mutation is 100% identical in vertebrates from mammals to fish. (B) Electrophoretic mobility shift assay (EMSA). Increasing concentrations (given in µM) of recombinant wild-type and mutant MITF protein from *E. coli* were incubated with a radioactively labeled double-stranded oligonucleotide and run on a non-denaturing polyacrylamide gel. The mutant MITF protein shows a weaker retention band than the wild-type MITF, which indicates a partially defective DNA binding activity (Table S3).

We sequenced the coding regions of six functional candidate genes in the macchiato stallion and his solid-colored parents. The investigated candidate genes are involved in white spotting (*KIT*, *MITF*) or coat color dilution (*MLPH*, *PMEL*, *SLC36A1*, and *SLC45A2*). We identified a *de novo* missense mutation in exon 6 of the *MITF* gene in the macchiato stallion (c.929A>G). We found the mutant allele at approximately 50% intensity in DNA samples from blood, hair roots and sperm, indicating that the macchiato stallion is not a mosaic. The mutant allele was not present in DNA from blood samples from either the mother or the father. The mutation affects a highly conserved amino acid of the basic DNA binding motif of the transcription factor MITF (p.N310S). We analyzed the DNA binding properties of the mutant MITF in an electrophoretic mobility shift assay and found that DNA binding activity was reduced by about 80%, probably through changes to the kinetics of binding (Figure 7, Table S3). A mutation at the same residue in the human MITF protein leads to Tietz syndrome, which is characterized by a more generalized depigmentation and profound obligate hearing loss compared to the slightly milder Waardenburg syndrome 2A, which is caused by many other mutations in the human *MITF* gene [25]. The MITF:p.N310S variant was absent from 96 solid-colored Franches-Montagnes horses.

## Discussion

As the splashed white phenotype is rather distinctive among the many different variations of white spotting phenotypes in horses, we started this investigation with the expectation of finding only a single causative mutation. However, the linkage/GWAS data suggested locus heterogeneity even within a family of closely related Quarter Horses. Using a positional candidate gene approach we subsequently identified three causative mutations for splashed white phenotypes and one causative mutation for another similar phenotype that we termed macchiato (Table 1).

Although we do not have functional proof for all of the mutations, we obtained sufficient ancillary evidence to claim their causality. For the *PAX3^C70Y^* allele the arguments are that it (1) occurs only in horses with the splashed white phenotype, (2) the *PAX3* locus was linked to this phenotype in a family, (3) mutations within the *PAX3* gene cause similar phenotypes in humans and mice, and (4) the mutation affects a highly conserved cysteine residue in the paired domain, that is predicted to participate in DNA binding. We were able to determine that this variant arose in 1987 and to identify the specific founder animal.

For the three *MITF* mutations the support is even better than for the *PAX3* mutation: (1) Multiple mutations within the same gene lead to comparable phenotypes, (2) the three variants occur exclusively in horses with characteristic depigmentation phenotypes, and (3) mutations within the *MITF* gene cause similar phenotypes in humans, mice, and dogs [18], [19], [24]. MITF^C280Sfs*20^ is the result of a frameshift mutation, which is extremely likely to affect the normal function of MITF as it truncates about half of the protein. The *MITF^prom1^* variant affects a region of the melanocyte-specific promoter that has been shown to function as a PAX3 binding site in humans and mice [20]–[22]. For *MITF^N310S^* we demonstrated that it represents a spontaneous *de novo* mutation in the macchiato horse that was born out of solid-colored parents free of this allele. We also provide functional evidence that the MITF^N310S^ protein has a reduced DNA binding capability. Taken together these data strongly argue for the causality of the four mutations in *PAX3* and *MITF* that we report in this study.

Our findings will be of relevance to horse breeders. The *MITF^prom1^* allele arose at least several hundred years ago, it is relatively common, and it occurs in several modern horse breeds. Horses homozygous for this allele are viable and typically have a more pronounced depigmentation phenotype than heterozygous horses. The *PAX3^C70Y^* allele is only 24 years old and occurs exclusively in Quarter and Paint Horses. We did not find a horse homozygous for this mutation, and based on data from mice it is unlikely that a homozygous *PAX3^C70Y/C70Y^* horse would be viable. As PAX3 is required for several key steps in neural development, homozygosity for this allele will most likely result in embryonic or fetal lethality [26]. Therefore, the mating of two heterozygous *PAX3* ^+/*C70Y*^ horses is not recommended in order to avoid the accidental production of an embryo homozygous for this allele. The MITF^C280Sfs*20^ and *MITF^N310S^* alleles are extremely rare. Data from mice again suggest that these alleles will most likely result in severe clinical phenotypes such as e.g. microphthalmia in the homozygous state [27]. Therefore, horses with such alleles should also not be mated to each other.

Depigmentation or white spotting phenotypes represent a complex trait, ranging from horses with a wild-type phenotype (no unpigmented skin areas) up to completely white horses. Previously, a number of single gene mutations in *KIT* and *EDNRB* have been shown to cause either dominant white or extreme white spotting phenotypes [10]–[17, Figure S5]. With this study we now report a series of mutations that lead to milder depigmentation phenotypes, which phenotypically overlap with the common white markings seen at the head and legs of many domestic horses. The newly reported mutations interact with other genetic factors. Chestnut horses carrying a mutation in *MITF* and/or *PAX3* show a more pronounced depigmentation phenotype than bay horses with the same mutations. Thus, the basic coat color or more specifically the genotype at *MC1R* modifies the phenotypic expression of the reported mutations. The correlation between chestnut coat color and increased size of white markings has been reported before [28], [29]. The detailed analysis of depigmentation phenotypes may help us to better understand complex genetic networks on a molecular and functional level. In such networks, non-coding regulatory mutations such as *MITF^prom1^* will probably play a major role and need to be taken into consideration.

In conclusion, we report four different mutations leading to splashed white or the new macchiato coat color phenotype in horses, which show similarities to the human Waardenburg or Tietz syndromes, respectively. This study highlights the potential of coat color genetics to gain molecular insights into complex regulatory gene networks.

## Materials and Methods

### Ethics statement

All animal work was conducted in accordance with the relevant local guidelines (Swiss law on animal protection and welfare - permit to the Swiss National Stud Farm no. 2227). The only animal experiment in our study was the collection of blood samples from horses by certified veterinarians.

### Animals and phenotypes

We analyzed a total of 239 horses, 106 animals with white spotting-phenotypes (cases) and 133 solid-colored controls. The cases consisted of 70 horses with the splashed white phenotype and another 36 horses with more extreme white spotting phenotypes (77 Quarter or American Paint Horses, 1 Hanoverian, 11 Icelandic Horses, 1 Miniature Horse, 2 Oldenburger, 1 Shetland Pony, 8 Thoroughbreds, 5 Trakehner). The 133 solid-colored controls consisted of 21 Quarter Horses, 1 American Standardbred, 96 Franches-Montagnes horses, 1 Haflinger, 4 Icelandic Horses, 7 Noriker, 1 Trakehner, and 2 European Warmblood horses. We considered Quarter Horses and American Paint Horses to represent one joint population as several horses in our study had double registrations and as it is quite common that offspring of Quarter Horse parents with white spotting phenotypes are registered as American Paint Horses.

We considered the proportion of white face area as the primary phenotypic criterion. To quantify this proportion we drew the heads of the horses in a standard perspective from photos (Figure S2, Figure S3). We then measured the proportion of unpigmented face area in comparison to a horse that we considered to have a 100% white face. Horses with ≥20% white face area were considered to have a white spotting phenotype. If a horse had ≥20% white face area and ≤10% white area on the body, it was considered “splashed white”. If a horse had ≥20% white face area and >10% white body area, it was considered to have an “other white spotting” phenotype. Horses with ≤3% white face area and 0% white body area were considered as solid-colored controls. Horses with a white face area ranging from 3–20% were classified as unknown phenotype.

### Linkage and association mapping

We isolated genomic DNA from either EDTA blood or hair root samples from all horses. We genotyped 31 horses with the illumina equine 50K SNP beadchip containing 54,602 SNPs. We used the PLINK v1.07 software for pruning of the genotype data set [30]. We removed 28,381 SNPs that did not have genotype calls in every animal and 10,753 SNPs that had minor allele frequencies below 5%. For the final analysis 31 horses and 19,319 SNPs remained. We used the Merlin software [31] and a fully dominant model of inheritance to analyze the data for parametric linkage. We used the PLINK software for genome-wide association analyses. Empirical genome-wide significance levels were determined by performing 100,000 random permutations of the assigned phenotypes.

### DNA sequencing and mutation analysis

We amplified exons, flanking intron regions, and proximal promoter sequences of the *KIT*, *MITF*, and *PAX3* genes as well as exon 2 of the *EDNRB* gene (Table S4). We subsequently sequenced the PCR products on an ABI 3730 capillary sequencer (Life Technologies). We analyzed sequencing data with the Sequencher 4.9 software for polymorphisms (Gene Codes). In the macchiato horse and its parents we sequenced all exons of the *KIT*, *MITF*, *MLPH*, *PMEL*, *SLC36A1*, and *SLC45A2* genes.

### Electrophoretic mobility shift assay (EMSA)

We expressed recombinant wild-type and N310S MITF from in *E. coli* Rosetta 2 cells in LB medium supplemented with 1% glucose (amino acid residues 112–207 from uniprot acc. Q95MD1). We lysed the cells by sonication in 20 mM Tris, 500 mM NaCl, 50 mM imidazole, 0.5 mM phenylmethylsulfonyl fluoride (PMSF), 0.1% (v/v) β-mercaptoethanol, pH 9. We applied the soluble fraction to Ni-NTA resin and eluted MITF with an imidazole step gradient (0.2–0.6 M) and further purified the proteins by reverse phase HPLC on a C18 column, using a gradient of 5–95% acetonitrile (0.1% TFA) over 20 ml at 1 ml/min. We lyophilised the protein-containing fractions and stored them at −20°C. The protein was refolded by resuspension in 20 mM Tris (pH 7.4), and we confirmed the correct folding by circular dichroism (CD) spectropolarimetry.

We performed the EMSA using an M-Box containing oligonucleotide (5′-GGAAAGTTAGTCATGTGCTTTTCAGAAGA-3′) as previously described [32]. The EMSA reactions contained 20 mM Tris, 50 mM NaCl, 5 mM MgCl_2_, 1 mM DTT, and 33 µg/ml BSA at pH 7.4. After incubation on ice for 30 min, we separated the samples on a 6% (w/v) non-denaturing polyacrylamide gel, in 0.5× TBE (45 mM Tris, 45 mM boric acid, 2.5 mM EDTA, pH 8.3) and analyzed the gels using a PhosphorImager (Molecular Dynamics). We quantified the bands from three repeated experiments and calculated average values and standard deviations.

## Supporting Information

Figure S1 Pedigree of a Quarter Horse family segregating for the splashed white phenotype. Horses with the splashed white phenotype are drawn as solid symbols. The 31 horses that were typed on the equine SNP chip are marked with asterisks. Sample numbers are shown next to horses, from which DNA samples were available. The genotypes of the *MITF^prom1^* and *PAX3^C70Y^* variants are indicated. The *PAX3^C70Y^* allele most likely arose *de novo* in the germline of the splashed white mare QH095. A hair sample of QH095 tested homozygous wildtype, whereas her two splashed white sons QH096 and QH140 both carry this allele. All tested non-splashed white horses of this family were homozygous wildtype for both the *MITF^prom1^* and the *PAX3^C70Y^* variant. All but two of the tested splashed white horses in this pedigree carried the *MITF^prom1^* and/or the *PAX3^C70Y^* variant. The remaining two splashed white horses, in which we could not identify a causative mutation, are QH082 and his mother QH084 in the lower left corner of this pedigree.
(TIF)

Figure S2 Quantification of the white face area. The heads of horses were re-drawn in a standard perspective from the available photographs. The area of the unpigmented skin on the face was then measured and expressed as % white face in relation to a horse considered to have 100% white face. Horses with ≥20% white face area were considered cases and horses with ≤3% white face area were considered solid-colored controls.
(TIF)

Figure S3 Standardized head views of splashed white Quarter Horses for quantitative estimation of the white face area.
(PDF)

Figure S4 Blair Athol, painting by Harry Hall. Blair Athol was a famous Thoroughbred stallion born in 1861. We analyzed modern Trakehner and Quarter Horses with the *MITF^prom1^* mutation who share this stallion as a common ancestor. The extremely large blaze of Blair Athol suggests that this horse also carried the *MITF^prom1^* allele. (Image courtesy of Rehs Galleries, Inc., NYC, www.rehs.com)
(JPG)

Figure S5 Phenotypes of overo spotted horses with the *EDNRB^I118K^* allele. The overo spotting pattern is quite variable in expression and typically involves much more extensive depigmentation on the body than the splashed white pattern. However, horses with minimal expression of overo spotting can have a very similar coat color phenotype as splashed white horses. (A) Horse with a typical overo spotting pattern. (B) Horse with a minimal overo spotting pattern. (C) Foal carrying a combination of *EDNRB^I118K^* and *MITF^prom1^*. The mare in the background is the same horse as in (B).
(TIF)

Table S1 Polymorphisms and genotypes.
(XLS)

Table S2 White face area in Quarter Horses with specific genotypes.
(XLS)

Table S3 Quantification of EMSA data for the MITF^N310S^ mutant protein.
(XLS)

Table S4 Primer sequences.
(XLS)
