# Correction: Mutations in *MITF* and *PAX3* Cause “Splashed White” and Other White Spotting Phenotypes in Horses

**DOI:** 10.1371/journal.pgen.1008321

**Published:** 2019-08-02

**Authors:** Regula Hauswirth, Bianca Haase, Marlis Blatter, Samantha A. Brooks, Dominik Burger, Cord Drögemüller, Vincent Gerber, Diana Henke, Jozef Janda, Rony Jude, K. Gary Magdesian, Jacqueline M. Matthews, Pierre-André Poncet, Vilhjálmur Svansson, Teruaki Tozaki, Lorna Wilkinson-White, M. Cecilia T. Penedo, Stefan Rieder, Tosso Leeb

There are errors in the identification of an allele, *PAX3*^*C70Y*^, arising by a *de novo* mutation event in a Quarter Horse mare born in 1987. The authors discovered a sample mix-up concerning the erroneously claimed Quarter Horse founder mare, labeled QH095 and genotyped *PAX3*^*+/+*^. Through analysis of an independent sample of QH095, the authors identified the genotype *PAX3*^*C70Y/+*^ in the new sample. Therefore, QH095 is not the founder animal for the *PAX3*^*C70Y*^ allele.

## Amendments to Results and Discussion Sections

In the ‘Splashed white in a Quarter Horse family’ subsection of the Results, the fifth and sixth sentences of the third paragraph are incorrect: “All these horses traced back to a female Quarter Horse born in 1987, whose genomic DNA from a hair-root sample tested homozygous wild-type. Thus, the mutation most likely arose in the germline of this animal.” Following reanalysis, the true genotype of this Quarter horse is *PAX3*^*C70Y/+*^.

The fourth sentence of the fourth paragraph within the Discussion is also incorrect: “The *PAX3*^*C70Y*^ allele is only 24 years old and occurs exclusively in Quarter and Paint Horses.” Due to the sample mix-up, the authors can no longer date the mutation event.

## Amendments to S1 Fig Legend

Parts of the fifth and sixth sentences of the legend are incorrect: “The *PAX3*^*C70Y*^ allele most likely arose *de novo* in the germline of the splashed white mare QH095. A hair sample of QH095 tested homozygous wildtype, whereas her two splashed white sons QH096 and QH140 both carry this allele.” Following reanalysis, the true genotype of this Quarter horse is *PAX3*^*C70Y/+*^ and the claim of a *de novo* mutation event is incorrect. The authors provide a corrected version below.

## Supporting information

S1 Fig Pedigree of a Quarter Horse family segregating for the splashed white phenotype. Horses with the splashed white phenotype are drawn as solid symbols. The 31 horses that were typed on the equine SNP chip are marked with asterisks. Sample numbers are shown next to horses, from which DNA samples were available. The genotypes of the *MITF*^*prom1*^ and *PAX3*^*C70Y*^ variants are indicated. The *PAX3*^*C70Y*^ allele most likely arose in an ancestor of the splashed white mare QH095. All tested non-splashed white horses of this family were homozygous wildtype for both the *MITF*^*prom1*^ and the *PAX3*^*C70Y*^ variant. All but two of the tested splashed white horses in this pedigree carried the *MITF*^*prom1*^ and/or the *PAX3*^*C70Y*^ variant. The remaining two splashed white horses, in which we could not identify a causative mutation, are QH082 and his mother QH084 in the lower left corner of this pedigree.
(DOCX)
