# Supplementary figures and images for: Mutations in MITF and PAX3 Cause “Splashed White” and Other White Spotting Phenotypes in Horses
Source: PLoS Genet. 2012 Apr 12;8(4):e1002653. doi: 10.1371/journal.pgen.1002653 (PMC3325211; doi:10.1371/journal.pgen.1002653)

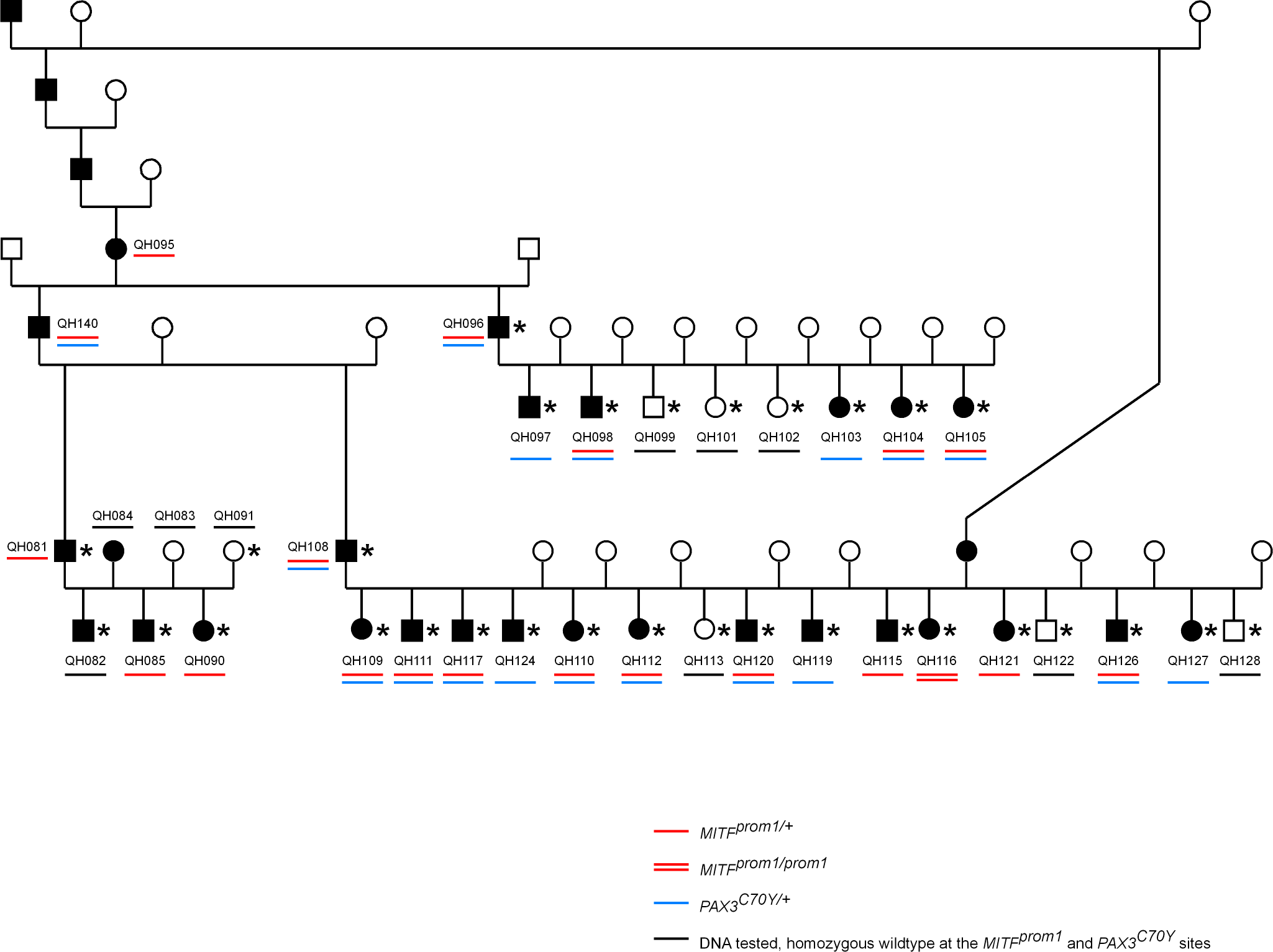

Supplement: Figure S1 — Pedigree of a Quarter Horse family segregating for the splashed white phenotype. Horses with the splashed white phenotype are drawn as solid symbols. The 31 horses that were typed on the equine SNP chip are marked with asterisks. Sample numbers are shown next to horses, from which DNA samples were available. The genotypes of the MITFprom1 and PAX3C70Y variants are indicated. The PAX3C70Y allele most likely arose de novo in the germline of the splashed white mare QH095. A hair sample of QH095 tested homozygous wildtype, whereas her two splashed white sons QH096 and QH140 both carry this allele. All tested non-splashed white horses of this family were homozygous wildtype for both the MITFprom1 and the PAX3C70Y variant. All but two of the tested splashed white horses in this pedigree carried the MITFprom1 and/or the PAX3C70Y variant. The remaining two splashed white horses, in which we could not identify a causative mutation, are QH082 and his mother QH084 in the lower left corner of this pedigree. (TIF) [file pgen.1002653.s001.tif]

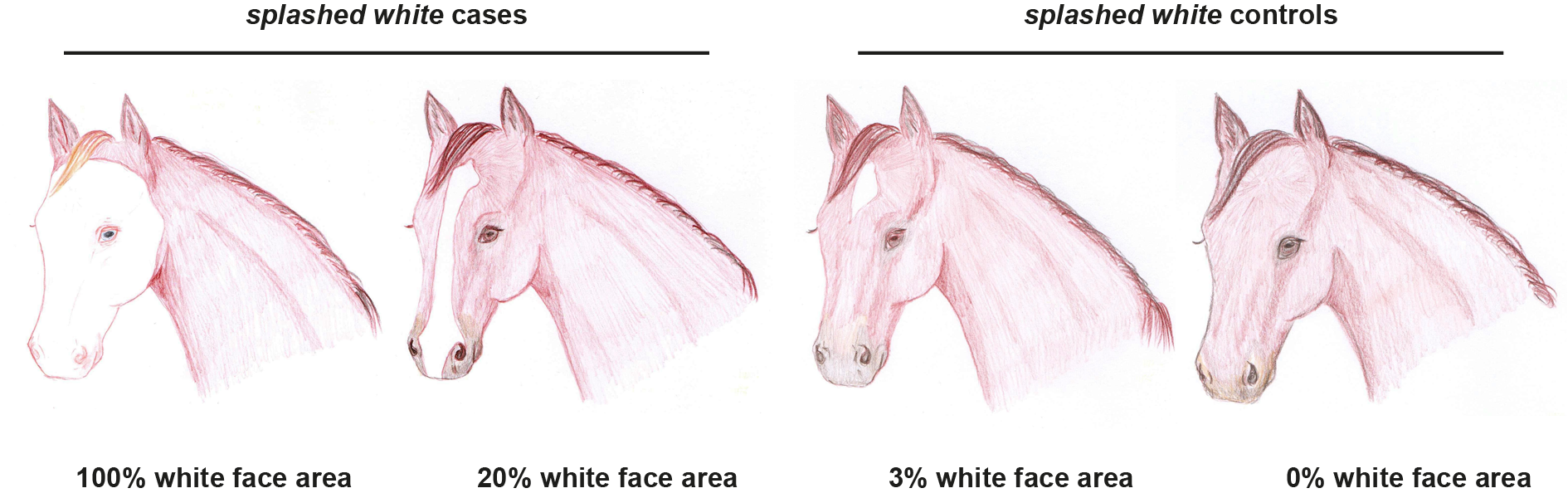

Supplement: Figure S2 — Quantification of the white face area. The heads of horses were re-drawn in a standard perspective from the available photographs. The area of the unpigmented skin on the face was then measured and expressed as % white face in relation to a horse considered to have 100% white face. Horses with ≥20% white face area were considered cases and horses with ≤3% white face area were considered solid-colored controls. (TIF) [file pgen.1002653.s002.tif]

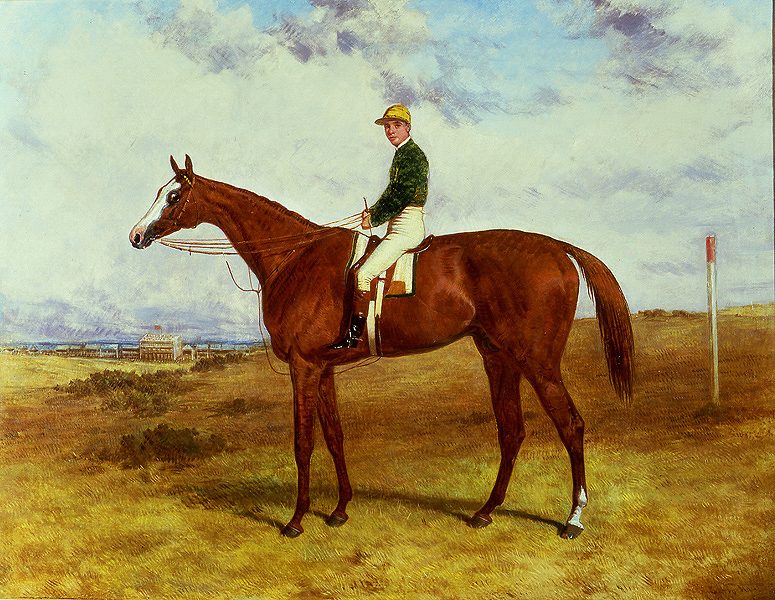

Supplement: Figure S4 — Blair Athol, painting by Harry Hall. Blair Athol was a famous Thoroughbred stallion born in 1861. We analyzed modern Trakehner and Quarter Horses with the MITFprom1 mutation who share this stallion as a common ancestor. The extremely large blaze of Blair Athol suggests that this horse also carried the MITFprom1 allele. (Image courtesy of Rehs Galleries, Inc., NYC, www.rehs.com) (JPG) [file pgen.1002653.s004.jpg]

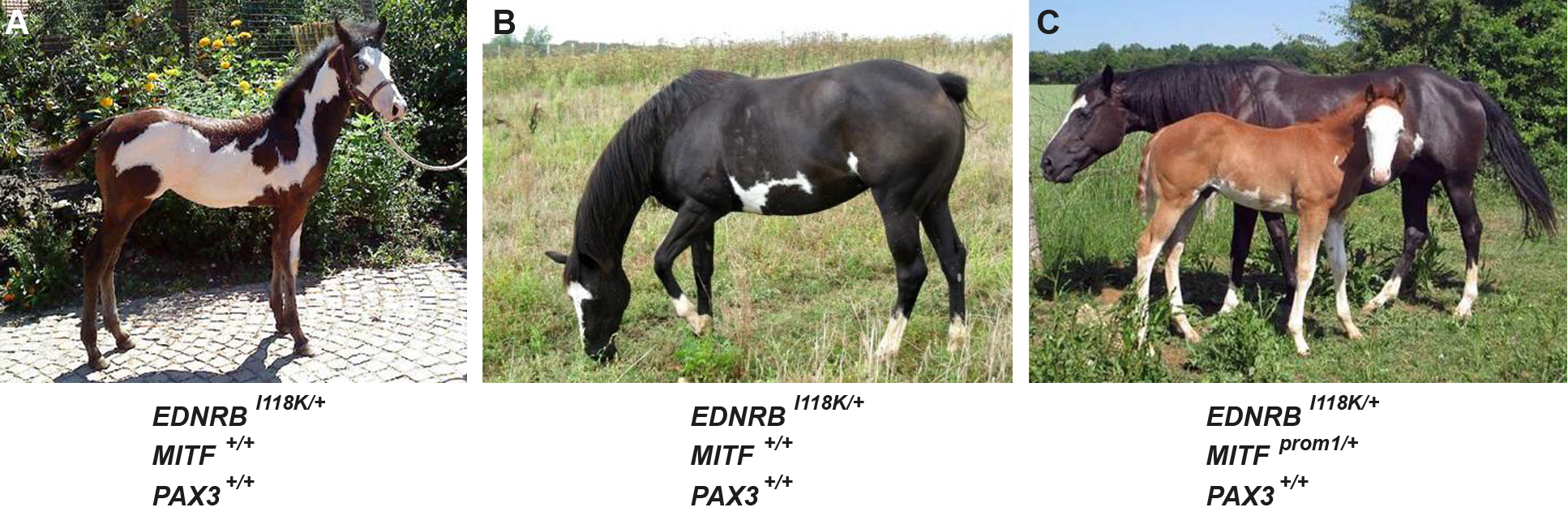

Supplement: Figure S5 — Phenotypes of overo spotted horses with the EDNRBI118K allele. The overo spotting pattern is quite variable in expression and typically involves much more extensive depigmentation on the body than the splashed white pattern. However, horses with minimal expression of overo spotting can have a very similar coat color phenotype as splashed white horses. (A) Horse with a typical overo spotting pattern. (B) Horse with a minimal overo spotting pattern. (C) Foal carrying a combination of EDNRBI118K and MITFprom1. The mare in the background is the same horse as in (B). (TIF) [file pgen.1002653.s005.tif]
