# Supplementary material for: Mutations in MITF and PAX3 Cause “Splashed White” and Other White Spotting Phenotypes in Horses
Source: PLoS Genet. 2012 Apr 12;8(4):e1002653. doi: 10.1371/journal.pgen.1002653 (PMC3325211; doi:10.1371/journal.pgen.1002653)
Supplement: Figure S3 — Standardized head views of splashed white Quarter Horses for quantitative estimation of the white face area. (PDF) [file pgen.1002653.s003.pdf]

Quantitative coat color phenotypes of horses with specific genotypes. White face areas are indicated.

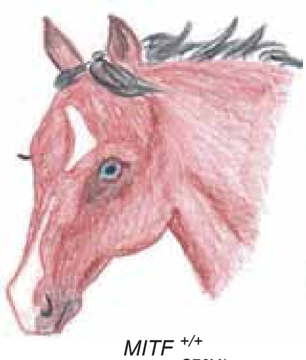

QH097
22%
MITF $^{+/+}$
PAX3 $^{C70Y/+}$
bay

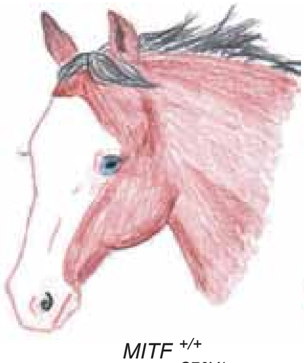

QH103
55%
MITF $^{+/+}$
PAX3 $^{C70Y/+}$
bay

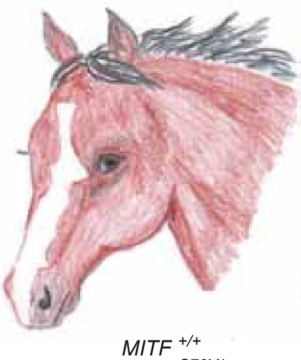

QH123
34%
MITF $^{+/+}$
PAX3 $^{C70Y/+}$
bay

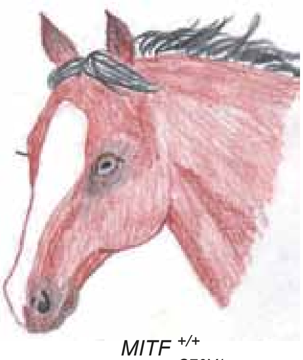

QH125
34%
MITF $^{+/+}$
PAX3 $^{C70Y/+}$
bay

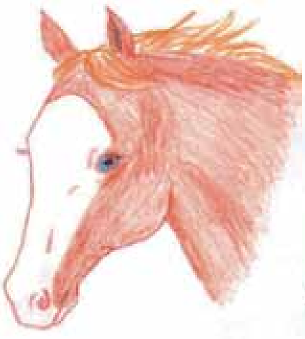

QH118
62%
MITF $^{+/+}$
PAX3 $^{C70Y/+}$
chestnut

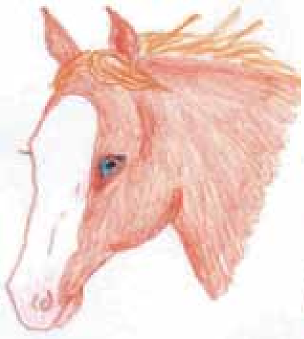

QH119
60%
MITF $^{+/+}$
PAX3 $^{C70Y/+}$
chestnut

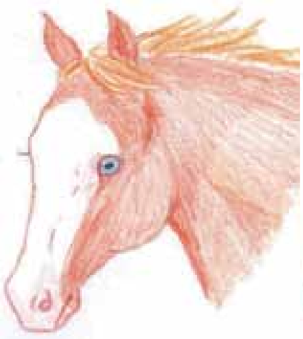

QH124
60%
MITF $^{+/+}$
PAX3 $^{C70Y/+}$
chestnut

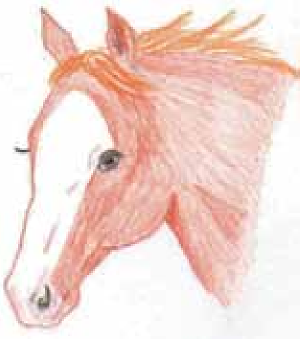

QH127
52%
MITF $^{+/+}$
PAX3 $^{C70Y/+}$
chestnut

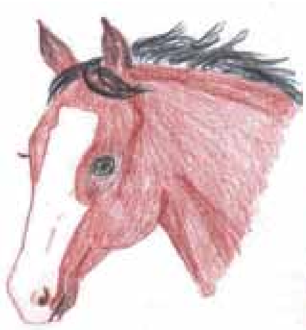

QH095
37%
MITF $^{prom1/+}$
PAX3 $^{+/+}$
bay

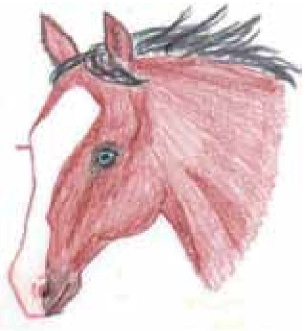

QH151
38%
MITF $^{prom1/+}$
PAX3 $^{+/+}$
bay

Quantitative coat color phenotypes of horses with specific genotypes. White face areas are indicated.

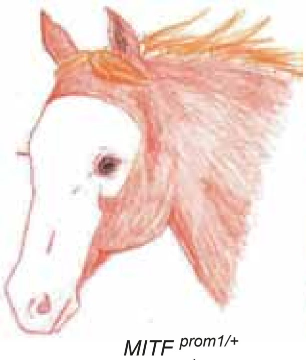

QH081 **76%** *MITF*$^{prom1/+}$ *PAX3*$^{+/+}$ chestnut

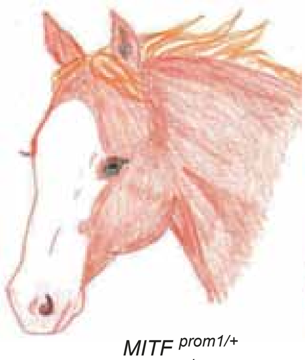

QH085 **54%** *MITF*$^{prom1/+}$ *PAX3*$^{+/+}$ chestnut

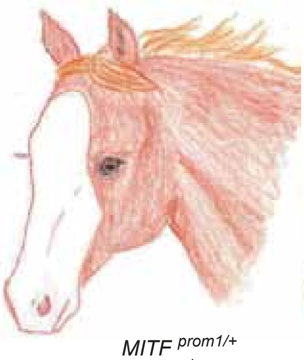

QH090 **57%** *MITF*$^{prom1/+}$ *PAX3*$^{+/+}$ chestnut

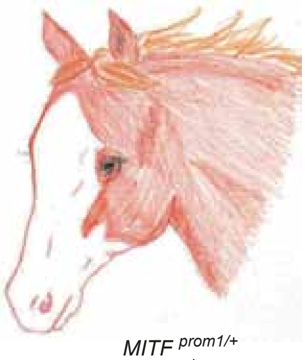

QH092 **62%** *MITF*$^{prom1/+}$ *PAX3*$^{+/+}$ chestnut

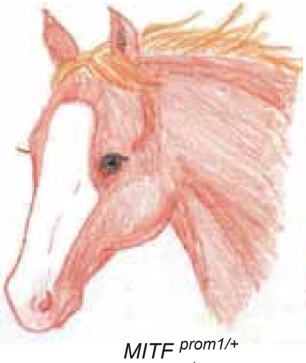

QH115 **45%** *MITF*$^{prom1/+}$ *PAX3*$^{+/+}$ chestnut

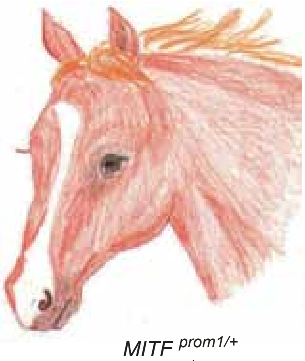

QH121 **21%** *MITF*$^{prom1/+}$ *PAX3*$^{+/+}$ chestnut

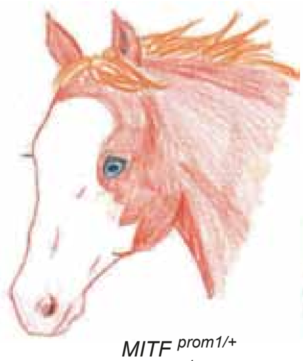

QH129 **72%** *MITF*$^{prom1/+}$ *PAX3*$^{+/+}$ chestnut

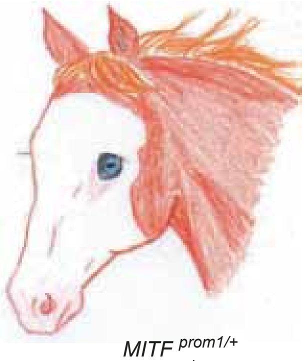

QH142 **90%** *MITF*$^{prom1/+}$ *PAX3*$^{+/+}$ chestnut

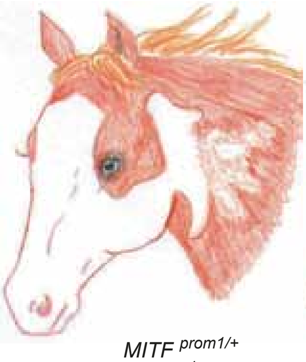

QH172 **78%** *MITF*$^{prom1/+}$ *PAX3*$^{+/+}$ chestnut

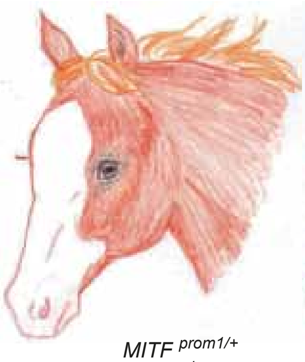

QH173 **62%** *MITF*$^{prom1/+}$ *PAX3*$^{+/+}$ chestnut

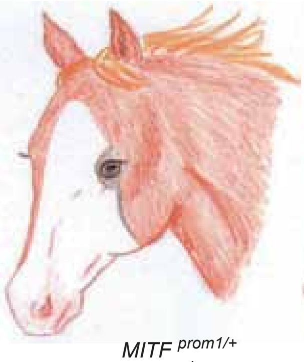

QH174 **53%** *MITF*$^{prom1/+}$ *PAX3*$^{+/+}$ chestnut

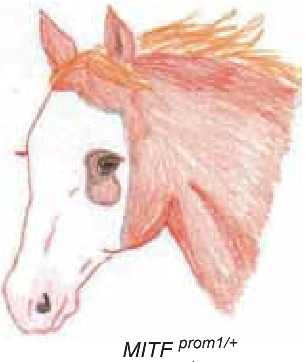

QH176 **71%** *MITF*$^{prom1/+}$ *PAX3*$^{+/+}$ chestnut

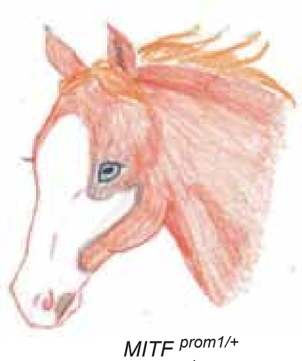

QH177 **56%** *MITF*$^{prom1/+}$ *PAX3*$^{+/+}$ chestnut

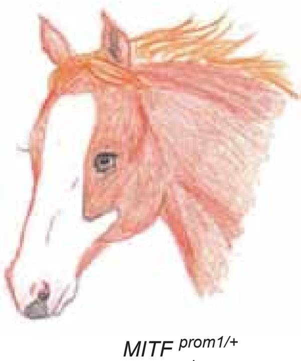

QH178 **51%** *MITF*$^{prom1/+}$ *PAX3*$^{+/+}$ chestnut

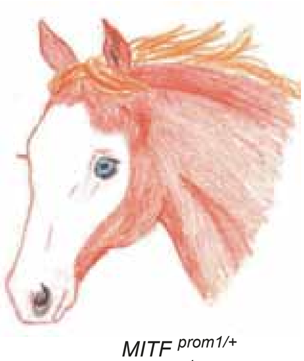

QH180 **71%** *MITF*$^{prom1/+}$ *PAX3*$^{+/+}$ chestnut

Quantitative coat color phenotypes of horses with specific genotypes. White face areas are indicated.

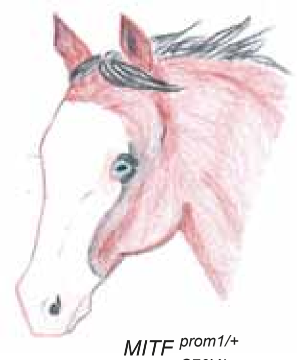

QH096
64%
$\mathit{MITF}^{prom1/+}$
$\mathit{PAX3}^{C70Y/+}$
bay

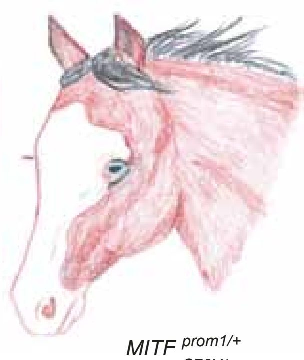

QH098
52%
$\mathit{MITF}^{prom1/+}$
$\mathit{PAX3}^{C70Y/+}$
bay

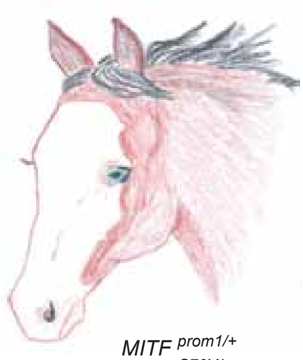

QH100
72%
$\mathit{MITF}^{prom1/+}$
$\mathit{PAX3}^{C70Y/+}$
bay

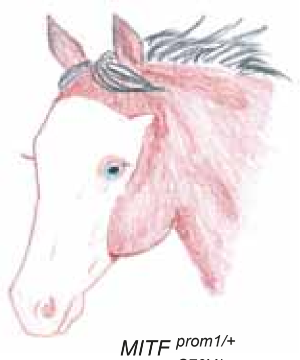

QH104
80%
$\mathit{MITF}^{prom1/+}$
$\mathit{PAX3}^{C70Y/+}$
bay

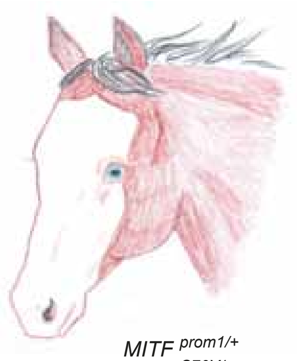

QH105
80%
$\mathit{MITF}^{prom1/+}$
$\mathit{PAX3}^{C70Y/+}$
bay

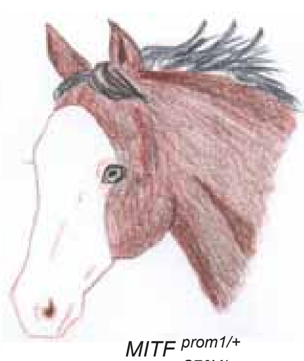

QH107
66%
$\mathit{MITF}^{prom1/+}$
$\mathit{PAX3}^{C70Y/+}$
dark bay

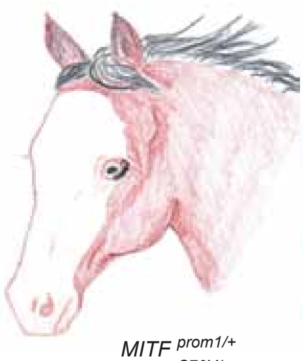

QH110
70%
$\mathit{MITF}^{prom1/+}$
$\mathit{PAX3}^{C70Y/+}$
bay

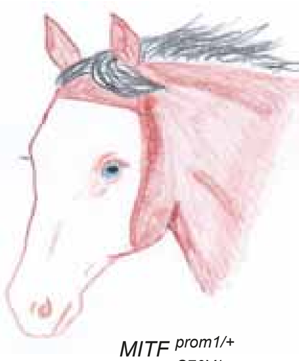

QH114
90%
$\mathit{MITF}^{prom1/+}$
$\mathit{PAX3}^{C70Y/+}$
bay

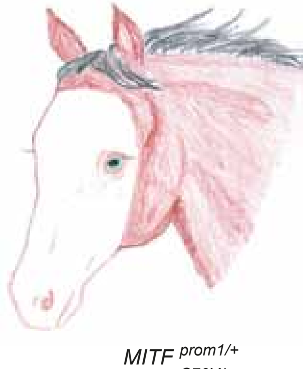

QH148
85%
$\mathit{MITF}^{prom1/+}$
$\mathit{PAX3}^{C70Y/+}$
bay

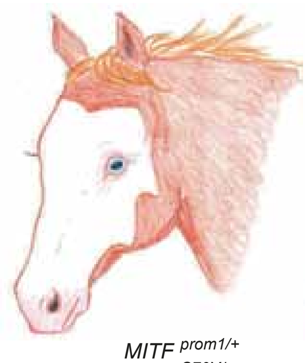

QH076
74%
$\mathit{MITF}^{prom1/+}$
$\mathit{PAX3}^{C70Y/+}$
chestnut

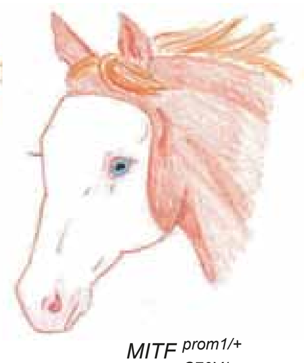

QH106
88%
$\mathit{MITF}^{prom1/+}$
$\mathit{PAX3}^{C70Y/+}$
chestnut

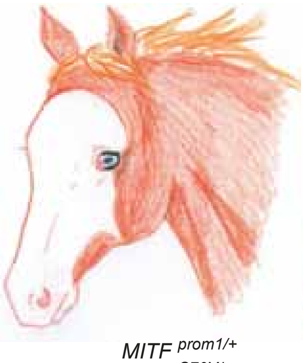

QH108
73%
$\mathit{MITF}^{prom1/+}$
$\mathit{PAX3}^{C70Y/+}$
chestnut

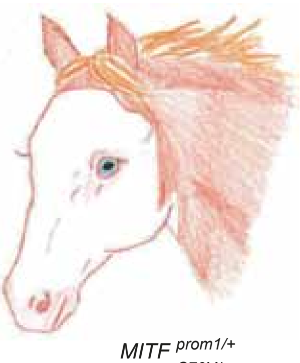

QH109
100%
$\mathit{MITF}^{prom1/+}$
$\mathit{PAX3}^{C70Y/+}$
chestnut

Quantitative coat color phenotypes of horses with specific genotypes. White face areas are indicated.

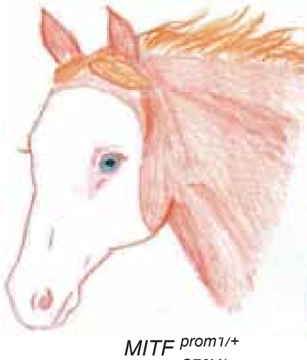

QH111
95%
MITF $^{prom1/+}$
PAX3 $^{C70Y/+}$
chestnut

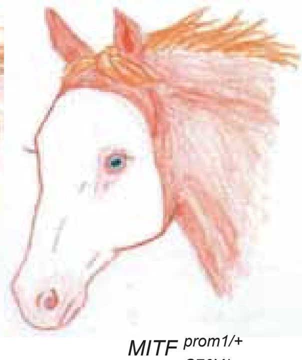

QH112
95%
MITF $^{prom1/+}$
PAX3 $^{C70Y/+}$
chestnut

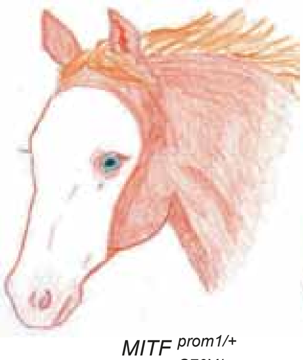

QH117
84%
MITF $^{prom1/+}$
PAX3 $^{C70Y/+}$
chestnut

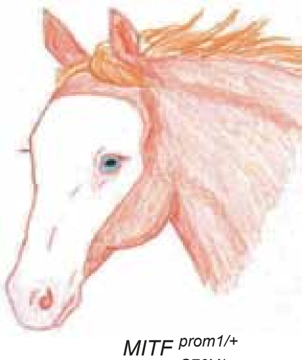

QH120
86%
MITF $^{prom1/+}$
PAX3 $^{C70Y/+}$
chestnut

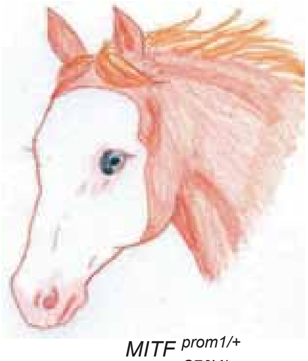

QH140
88%
MITF $^{prom1/+}$
PAX3 $^{C70Y/+}$
chestnut
