# Supplementary material for: Correction: Mutations in MITF and PAX3 Cause “Splashed White” and Other White Spotting Phenotypes in Horses
Source: PLoS Genet. 2019 Aug 2;15(8):e1008321. doi: 10.1371/journal.pgen.1008321 (PMC6677290; doi:10.1371/journal.pgen.1008321)
Supplement: S1 Fig — Horses with the splashed white phenotype are drawn as solid symbols. The 31 horses that were typed on the equine SNP chip are marked with asterisks. Sample numbers are shown next to horses, from which DNA samples were available. The genotypes of the MITFprom1 and PAX3C70Y variants are indicated. The PAX3C70Y allele most likely arose in an ancestor of the splashed white mare QH095. All tested non-splashed white horses of this family were homozygous wildtype for both the MITFprom1 and the PAX3C70Y variant. All but two of the tested splashed white horses in this pedigree carried the MITFprom1 and/or the PAX3C70Y variant. The remaining two splashed white horses, in which we could not identify a causative mutation, are QH082 and his mother QH084 in the lower left corner of this pedigree. (DOCX) [file pgen.1008321.s001.docx]

**Corrected Version with changes highlighted**


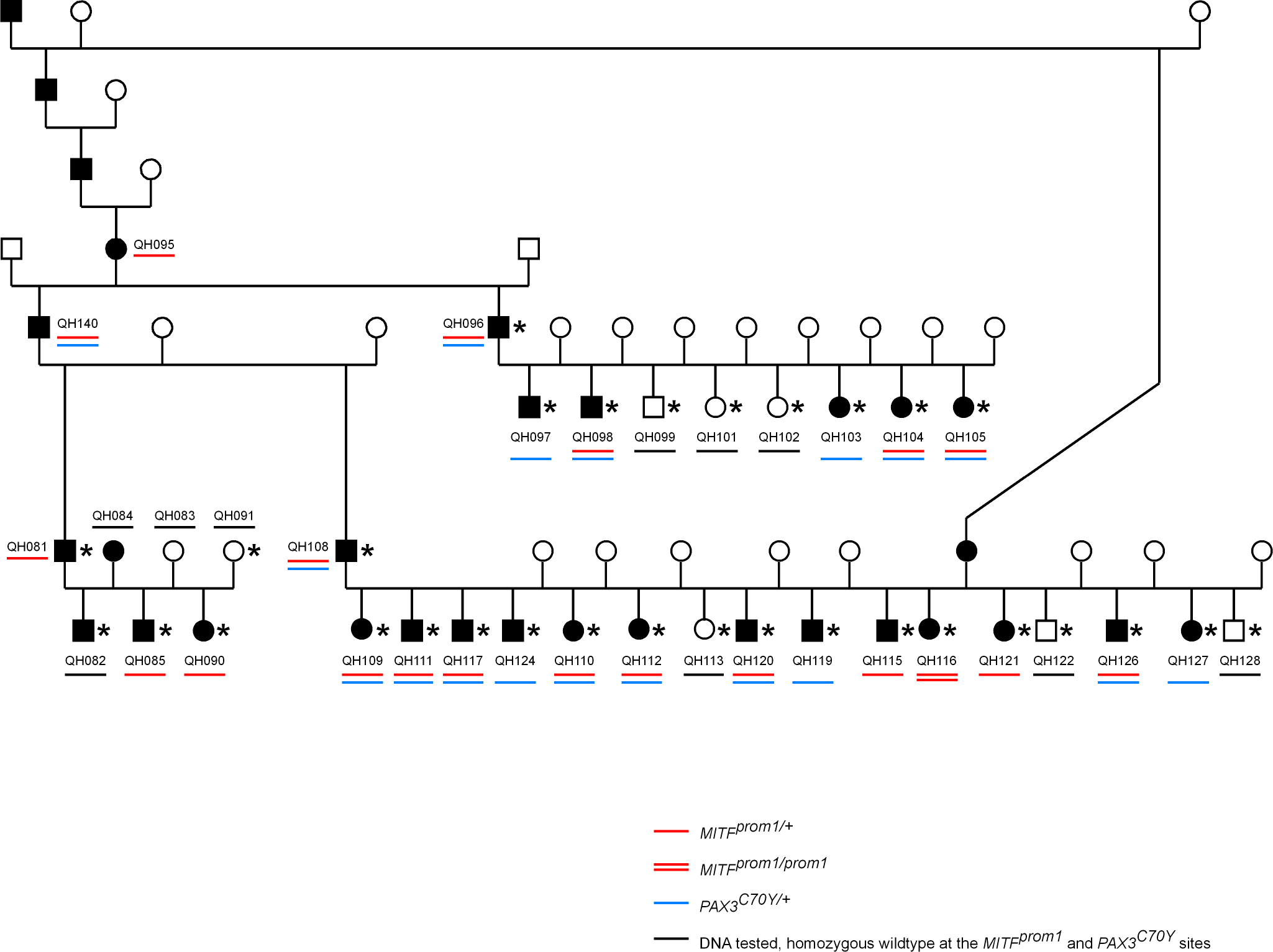


**Figure S1.**

Pedigree of a Quarter Horse family segregating for the splashed white phenotype. Horses with the splashed white phenotype are drawn as solid symbols. The 31 horses that were typed on the equine SNP chip are marked with asterisks. Sample numbers are shown next to horses, from which DNA samples were available. The genotypes of the *MITF^prom1^* and *PAX3^C70Y^* variants are indicated. The *PAX3^C70Y^* allele most likely arose in an ancestor of the splashed white mare QH095. All tested non-splashed white horses of this family were homozygous wildtype for both the *MITF^prom1^* and the *PAX3^C70Y^* variant. All but two of the tested splashed white horses in this pedigree carried the *MITF^prom1^* and/or the *PAX3^C70Y^* variant. The remaining two splashed white horses, in which we could not identify a causative mutation, are QH082 and his mother QH084 in the lower left corner of this pedigree.
